# Synthesis and Binding Ability of Molecular Probes Based on a Phenanthroline Derivative: Theory and Experiment

**DOI:** 10.3390/molecules181214840

**Published:** 2013-12-03

**Authors:** Xuefang Shang, Yingling Wang, Xiaofang Wei, Zhiyuan Fu, Jinlian Zhang, Xiufang Xu

**Affiliations:** 1 Department of Chemistry, Xinxiang Medical University, Xinxiang 453003, Henan, China; 2 School of Pharmacy, Xinxiang Medical University, Xinxiang 453003, Henan, China; 3 Department of Chemistry, Nankai University, Tianjin 300071, China

**Keywords:** synthesis, fluorescent probe, colorimetric recognition, theoretical investigation, phenanthroline derivative

## Abstract

A fluorescent and colorimetric molecular probe containing phenol groups has been designed and synthesized. The anion binding ability was evaluated for biolgically important anions (F^−^, Cl^−^, Br^−^, I^−^, AcO^−^ and H_2_PO_4_^−^) by theoretical investigation, UV-Vis and fluorescence spectroscopy and ^1^H-NMR titration experiments. Results indicated the probe showed strong binding ability for H_2_PO_4_^−^ without the interference of other anions tested and the interaction process was accompanied by color changes. Theoretical investigation analysis revealed that intramolecular hydrogen bonds existed in the structure of the probe and the roles of molecular frontier orbitals in molecular interplay were determined.

## 1. Introduction

In recent years, increasing attention in the field of host-guest chemistry has been devoted to the fast development of anion recognition system [1,2,3,4,5,6,7,8,9,10,11]. The development of fluorescent and colorimetric probes for anions is timely and an area of current interest. Water-soluble anions such as fluoride, chloride, bromide, dihyphosphate, *etc*. play crucial roles in a range of biological phenomena and are implicated in many disease states [12]. The importance of anions in biological systems, catalysts and environmental concerns necessitate the development of highly sensitive anion probes [13,14,15,16,17]. So far, the focus has been mainly on the design of a receptor which has the ability to recognize and bind biological anions. However, this approach suffers from shortcomings such as the high cost of synthesis and the loss of the real-time response. Therefore, the challenge faced by chemists becomes one of the detecting and amplifying an anion binding event to produce a measurable output for the further application of anion recognition. Furthermore, a well defined probe should be achieved by the coupling of selective bonding sites and signaling subunits.

**Scheme 1:**
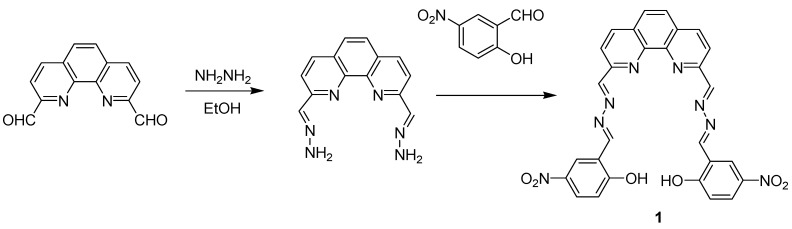
Synthesis route of compound **1**.

In order to make good use of fluorescent and colorimetric dual sensing molecules for the detection of H_2_PO_4_^−^, here we reported a phenanthroline derivative, 2,9-di((2',3'-diaza-4'-(2″-hydroxy-5″- nitrophenyl)-1',3'-butadiene)-1,10-phenanthroline (**1**, Scheme 1), which consists of a phenanthroline system and a nitro group as well as hydroxyl groups as a dual responsive fluorescent and colorimetric probe. The host–guest complexation for sensing H_2_PO_4_^−^ were investigated through UV-Vis, fluorescence spectroscopy and ^1^H-NMR measurements as well as theoretical investigation. 

## 2. Results and Discussion

### 2.1. UV-Vis Titration

**Figure 1:**
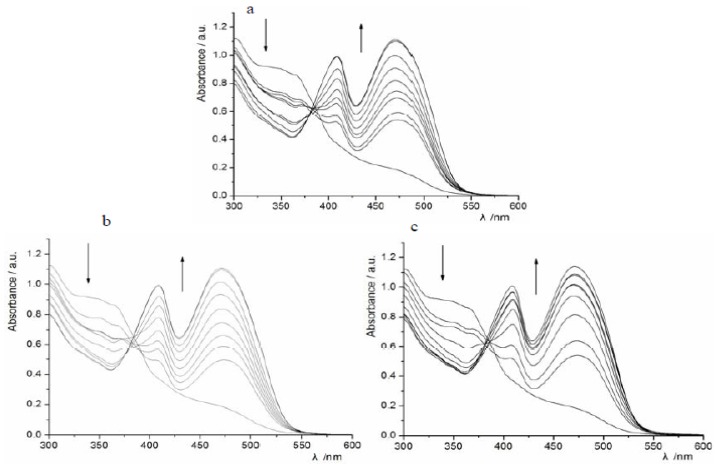
UV-vis spectral changes of compound **1** (4.0 × 10^−5^ mol·L^−1^**)** upon the addition of anions (0–160 × 10^−5^ mol·L^−1^), (**a**) H_2_PO_4_^−^; (**b**) AcO^−^, (**c**) F^−^. Arrows indicate the direction of increasing anion concentration.

**Figure 2:**
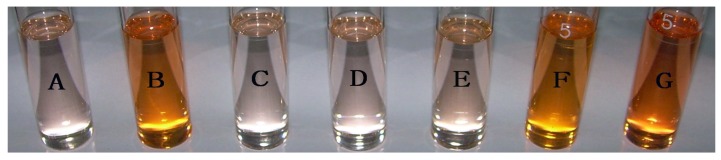
Color changes of compound **1** (4.0 × 10^−5^ mol·L^−1^) in DMSO in the absence and presence of different anions: (A: only compound **1**; B: compound **1** + 10 equiv. F^−^; C: compound **1** + 10 equiv. Cl^−^; D: compound **1** + 10 equiv. Br^−^; E: compound **1** + 10 equiv. I^−^; F: compound **1** + 10 equiv. AcO^−^; G: compound **1** + 10 equiv. H_2_PO_4_^−^).

The concomitant changes in the UV–Vis spectra of **1** upon the addition of H_2_PO_4_^−^ can be seen in Figure 1. Compound **1** exhibited an absorption band centered at 340 nm. As the concentration of H_2_PO_4_^−^ was increased, the absorption intensity at 340 nm gradually decreased and two new absorption peaks appeared at 400 nm and 475 nm, which was accompanied by a visual color change from light yellow to orange (Figure 2). Results indicated compound **1** interacted with H_2_PO_4_^−^ and new complex (**1**-H_2_PO_4_^−^) formed. However, the presence of a protic solvent such as H_2_O, which will compete with anions for binding sites and disturb the H-bond interactions between the host and the anionic guest, will lead to a reversal of the visual color change and the spectral changes [18,19]. Similar effects were observed in the UV–Vis spectra of **1** upon the addition of F^−^ or AcO^−^ ions (Figure 1). In the case of weak basic ions such as Cl^−^, Br^−^ and I^−^, the spectral changes were too small to calculate the corresponding binding constants.

### 2.2. Fluorescence Response

**Figure 3:**
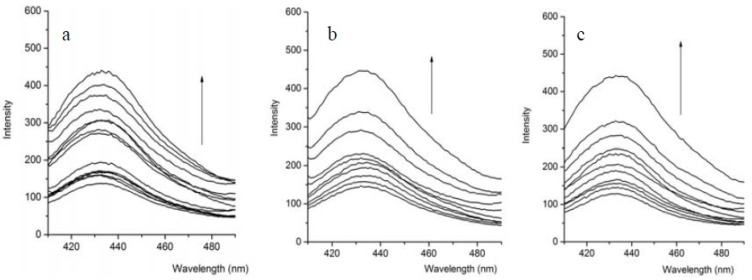
Changes in the emission spectra of compound **1** (4.0 × 10^−^^5^ mol·L^−1^) upon the addition of anions (0–80 × 10^−5^ mol·L^−1^), λ_ex_ = 284 nm, (**a**) H_2_PO_4_^−^; (**b**) AcO^−^, (**c**) F^−^. Arrows indicate the increased direction of anion additions.

The photophysical responses of compound **1** toward addition of the tested anions were also investigated. Just as Figure 3 shows, compound **1** exhibited an emission peak centered at 430 nm. Upon the addition of H_2_PO_4_^−^ to the solution of compound **1**, the fluorescence emission was strengthened, which showed that H_2_PO_4_^−^ interacted with the phenol O–H protons through hydrogen bonds. The photoinduced electronic transfer (PET) mechanism may explain the fluorescent increase [20,21,22,23]. Without the addition of H_2_PO_4_^−^, the hydrogen atoms of free compound **1** could form an intramolecular hydrogen bond with the nitrogen atom of the Schiff base, which leads to a photoinduced electron transfer and the fluorescence was strengthened. Upon addition of H_2_PO_4_^−^ to the solution, the electron transfer from compound **1** to the fluorophore became more feasible and the emission intensity was increased. Similar spectra changes were observed upon the addition of F^−^ or AcO^−^ (Figure 3). On the other hand, no significant spectral changes were observed upon the titration of **1** with Cl^−^, Br^−^, I^−^, signifying that compound **1** showed insignificant binding ability toward these anions.

### 2.3. Binding Constant

The spectral changes could be ascribed to the formation of 1:1 host-guest complexes according to a Job-plot curve. The obtained binding constants obtained using the method of non-linear least squares calculation according to the UV–Vis and fluorescence data are listed in Table 1 [24,25,26]. The binding ability trend of the probe to anions followed the order of: H_2_PO_4_^−^ > F^−^ > AcO^−^ >> Cl^−^ ~ Br^−^ ~ I^−^. It was apparent that the binding ability for anion can be rationalized on the basis of the anion’s basicity and the interactions between the host and the anionic guests. However, multiple hydrogen-bond interactions were also necessary in high-affinity anion binding sites. In addition, the tetrahedral configuration of H_2_PO_4_^−^ ion may match well the compound in terms of shape and could form multiple hydrogen bonding interactions (proved by theoretical investigation). Consequently, H_2_PO_4_^−^ ion can be strongly bound based on its binding constant. Moreover, the binding constants obtained by UV-Vis data were on the same order of magnitude as those obtained from fluorescence data, which indicated the results of binding constants obtained by UV-Vis data were corroborated by the fluorescence results.

**Table 1:** Binding constants of probe **1** with various anions.

Anion ^a^		K_s_(1)
H_2_PO_4_^−^	Absorption	(1.01 ± 0.08) × 10^5^
	Emission	(1.67 ± 0.22) × 10^5^
AcO^−^	Absorption	(3.43 ± 0.04) × 10^4^
	Emission	(2.89 ± 0.16) × 10^4^
F^−^	Absorption	(7.88 ± 0.11) × 10^4^
	Emission	(6.57 ± 0.35) × 10^4^

^a^ All anions were added in the form of tetra-*n*-butylammonium (TBA) salts.

### 2.4. 1H-NMR Titration

**Figure 4:**
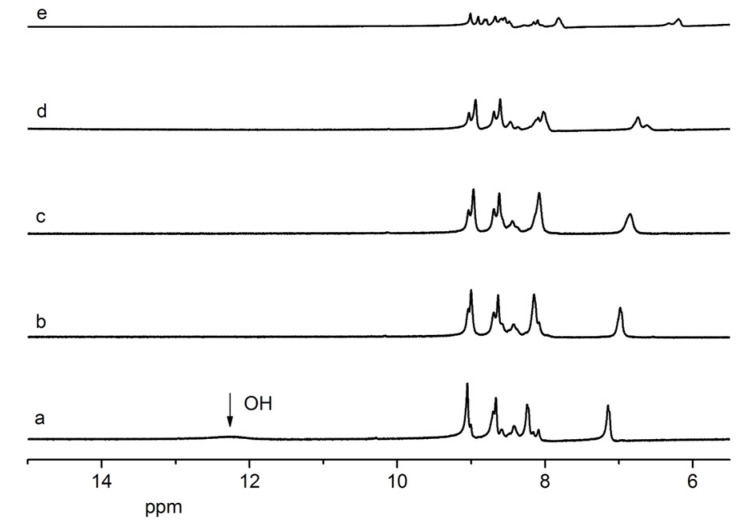
Portion of ^1^H-NMR spectra of compound **1** (0.02 mol·L^−1^) in DMSO-*d*_6_ in the presence of increasing amount of H_2_PO_4_^−^: (**a**) free **1**; (**b**) **1** + 0.5 equiv; (**c**) **1** +1.0 equiv; (d) **1** + 2.0 equiv; (e) **1** + 5.0 equiv. of [(n-Bu)_4_N] H_2_PO_4_.

In order to look into the anion binding properties, ^1^H-NMR titration of compound **1** with H_2_PO_4_^−^ was conducted as an example. Free compound **1** displayed one peak at 12.38 ppm in the downfield region which was attributed to –OH (Figure 4). Upon the stepwise addition of H_2_PO_4_^−^, the –OH signals weakened, gradually shifted to the downfield and completely disappeared. At the same time, a remarkable upfield shift of the aromatic protons (7.14 ppm) was observed, while the phenanthroline proton signals (8.66–8.09 ppm) almost did not move. The above observation indicated compound **1** interacted with H_2_PO_4_^−^ via hydrogen bonds and the interaction sites were the –OH groups. After H-bonding formed, a shielding effect existed in the region of –OH and H_2_PO_4_^−^. Then, the proton peak of –OH shifted downfield and disappeared completely. Meanwhile, for non-interacting sites like the aromatic rings. the deshielding effect was enhanced and their protons shifted in the upfield direction. The formation of hydrogen bonds changed the ICT properties of compound **1** and ultimately resulted in the observed color and spectral changes.

### 2.5. Theoretical Investigation

**Figure 5:**
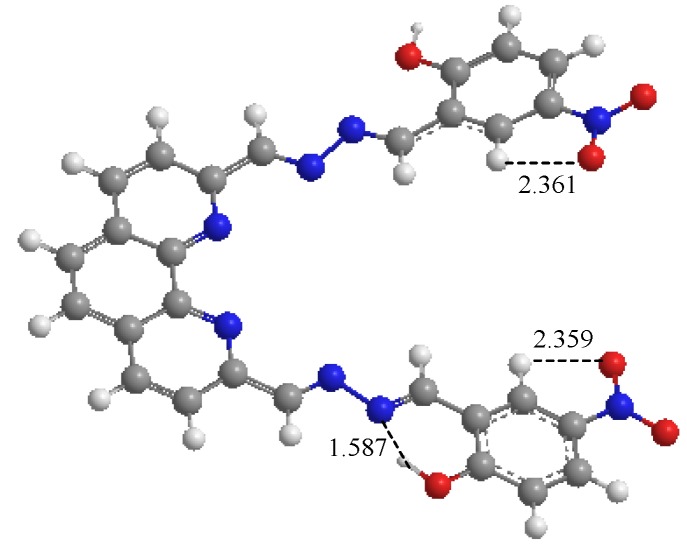
Optimized structure of compound **1**.

The geometry of compound **1** was optimized (Figure 5) using the Hartree–Fock (HF) method with the 3–21G basis sets. The calculation was performed with the Gaussian03 program [27].

From Figure 5, the distance of hydrogen atom in one interaction site (-OH) with the nearby nitrogen atom was 1.587 Å in compound **1**. This phenomenon showed an intramolecular hydrogen bond existed in the compound and a stable six-member cycle was formed. In addition, an intramolecular hydrogen bond also existed between the oxygen atom of –NO_2_ and a nearby hydrogen atom. Therefore, the binding ability of the oxyanion, H_2_PO_4_^−^, with compound **1** was the strongest among the anions tested.

**Figure 6:**
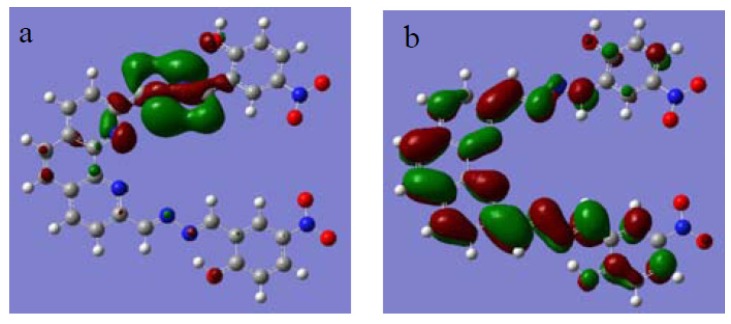
Molecular orbital levels of compound **1**: (**a**) HOMO; (**b**) LUMO.

In addition, selected frontier orbitals for compound **1** are shown in Figure 6. We introduce the molecular frontier orbitals in order to explain UV-Vis absorption spectra in the host–guest interaction process by electron transitions of frontier orbitals. The highest occupied obital (HOMO) density in compound **1** was mainly localized on the hydrazine moiety, while the lowest unoccupied orbital (LUMO) density was localized on the whole molecule, which demonstrated it was the electron transition of HOMO that gave rise to the red shift phenomenon observed in the UV-Vis spectra of the host-guest complex.

## 3. Experimental

### General

Most of the starting materials were obtained commercially and all reagents and solvents used were of analytical grade. 2,9-Dicarbaldehyde-1,10-phenanthroline and all anions, in the form of tetrabutylammonium salts (such as (*n*-C_4_H_9_)_4_NF, (*n*-C_4_H_9_)_4_NCl, (*n*-C_4_H_9_)_4_NBr, (*n*-C_4_H_9_)_4_NI, (*n*-C_4_H_9_)_4_NAcO, (*n*-C_4_H_9_)_4_NH_2_PO_4_), were purchased from Sigma-Aldrich Chemical Co. (Shanghai, China), and stored in a desiccator containing self-indicating silica under vacuum, and used without any further purification. Tetra-*n*-butylammonium salts were dried for 24 h in vacuum with P_2_O_5_ at 333 K before using. Dimethyl sulfoxide (DMSO) was distilled *in vacuo* after drying with CaH_2_. C, H, N elemental analysis were made on a Vario-EL instrument. ^1^H-NMR spectra were recorded on a UNITY Plus-400 MHz spectrometer. ESI-MS were performed with a MARINER apparatus. UV-Vis spectroscopy titration was made on a Shimadzu UV2550 spectrophotometer at 298 K. Fluorometric titration was performed on a Cary Eclipse fluorescence spectrophotometer at 298 K. The binding constant K_s_ was obtained by non-linear least square calculation method for data fitting. Compound **1** was synthesized according to the route shown in Scheme 1.

*2,9-Dimethylenehydrazine-1,10-phenanthroline*. 1,10-Phenanthroline-2,9-dicarbaldehyde (1 mmol, 236 mg) in dry ethanol (15 mL) was added to the ethanol (30 mL) solution containing hydrazine hydrate (80%, 0.5 mL) under stirring. Then the mixture was heated under refluxing for 8 h and the yellow precipitate was separated by filtration. The solid was washed with diethyl ether and dried under vacuum. Yield: 81%. ^1^H-NMR (400 MHz, DMSO-*d*_6_, 298 K) δ 9.52 (s, 4H, NH_2_), 8.49 (d, 2H, phen-H), 8.02 (d, 2H, phen-H), 7.67(d, 2H, phen-H), 7.53 (s, 2H, CH). Elemental analysis: Calc. for C_14_H_12_N_6_: C, 63.62; H, 4.58; N, 31.80; Found: C, 63.93; H, 4.36; N, 31.57. ESI-MS (*m/z*): 263.3 (*M*−H)^−^.

*2,9-di((2',3'-Diaza-4'-(2″-hydroxyl-3″-nitrophenyl)-1',3'-butadiene)-1,10-phenanthroline* (**1**). 2,9-Dimethylenehydrazine-1,10-phenanthroline (1 mmol, 264 mg) and 2-hydroxy-5-nitro-benzaldehyde (2 mmol, 334 mg) were suspended in dry ethanol (40 mL). The mixture was refluxing for 12 h and the yellow precipitate was separated by filtration. The solid was washed with diethyl ether and dried under vacuum. Yield: 77%. ^1^H-NMR (400 MHz, DMSO-*d*_6_, 298 K) δ12.38 (s, 2H, OH), 9.05 (s, 4H, CH), 8.66–8.09 (m, 6H, phen-H), 7.14 (d, 2H, ph-H). Elemental analysis: Calc. for C_28_H_18_N_8_O_6_: C, 59.79; H, 3.23; N, 19.92; Found: C, 60.02; H, 3.16; N, 20.27. ESI-MS (*m/z*): 561.0 (*M*−H)^−^.

## 4. Conclusions

In conclusion, a colorimetric molecular probe bearing phenanthroline and phenol groups was successfully designed and synthesized. The probe showed a strong binding ability for H_2_PO_4_^−^ without interference from other anions tested. The possible interaction mechanism was also researched by theoretical investigation, which demonstrated it was the electron transition of the HOMO that gave rise to the red shift phenomenon seen in the UV-Vis spectra of the host-guest complex. The combination of theory and experiment could provide a reliable base for the further application of the molecular probe.
